# ARRB1 Drives Gallbladder Cancer Progression by Facilitating TAK1/MAPK Signaling Activation

**DOI:** 10.7150/jca.53325

**Published:** 2021-01-30

**Authors:** Xudong Zhang, Zhijun Kong, Xiaoliang Xu, Xiao Yun, Jiadeng Chao, Dong Ding, Tao Li, Yuan Gao, Naifu Guan, Chunfu Zhu, Xihu Qin

**Affiliations:** 1 Department of Hepato-biliary-pancreatic Surgery, The Affiliated Changzhou No. 2 People's Hospital of Nanjing Medical University, XingLong Road 29#, Changzhou, Jiangsu 213000, P.R. China.; 2 Nanjing Medical University, Jiangsu 210000, P.R. China.; 3 Department of Hepatobiliary Surgery of Nanjing Drum Tower Hospital, Nanjing Medical University, Jiangsu 210000, China.

**Keywords:** ARRB1, TNF-α, TAK1, Gallbladder carcinoma.

## Abstract

Gallbladder carcinoma (GBC) is the most common malignancy of the biliary tract, with a dismal 5-year survival of 5%. Recently, ARRB1, as a molecular scaffold, has been proposed to participate in the progression of multiple malignancies. However, the effect and regulatory mechanisms of ARRB1 in GBC have not been investigated. Our study aimed to explore the biological functional status and the possible molecular mechanisms of ARRB1 with respect to GBC progression. The survey showed that human GBC tissues exhibited increased levels of ARRB1 compared with normal tissues, and the high expression of ARRB1 was associated with poor prognosis of GBC patients. A series of *in vitro* and *in vivo* functional experiments based on knockdown of ARRB1 uncovered that ARRB1 enhanced GBC cell proliferation, migration, and invasion. Furthermore, we reported that TAK1, a component of the TNF /MAPK pathway, is a vital downstream effector of ARRB1. In addition, siTAK1 could abolish the functional changes between ARRB1 overexpression GBC cells and control ones. Our data revealed that ARRB1 facilitated the carcinogenesis and development of GBC through TNF/TAK1/MAPK axis, suggesting that ARRB1 may be a promising biomarker and treatment target for GBC patients.

## Introduction

Gallbladder carcinoma (GBC) is the fifth most common gastrointestinal malignancy worldwide, with extremely poor prognosis 1. Radical cholecystectomy is still the main therapeutic schedule for GBC. However, only fewer than 10% of GBC patients are suitable for surgical excision due to the delayed diagnosis 2. For those unresectable GBC patients, the approved chemotherapy is only a palliative therapy method with low efficacy 3, 4. The etiology of GBC is intricate and multifactorial. Various risk factors associated with the development of GBC include geographical distribution, genetic susceptibility, race, older age, gender, gallstone (size > 3 cm) and chronic inflammation 5, 6. In view of the few therapeutic options, high recurrence and inadequate understanding of disease mechanisms, it is necessary to further study the mechanisms of GBC pathogenesis, explore new sensitive molecular biomarkers and screen more effective treatment targets for prolonging the survival.

β-arrestin 1(ARRB1), as a well-known primary effector of the GPCR pathway, has been shown to promote several stages in the progression of different cancers, including leukaemia, breast cancer, lung cancer, colon cancer and laryngeal carcinoma 7. As a molecular scaffold, ARRB1 could regulate cellular function by interacting with other partner proteins. For ovarian cancer, ARRB1 is activated by ET-1R and cooperates with p300 to participate in the interaction between HIF-1α, which enhances the transcription of genes required for tumor cell invasion and angiogenesis 8. Furthermore, ARRB1 is a potential prognostic biomarker for lung cancer and could predict the response to the chemotherapy of EGFR inhibitor 9. Our previous study has shown that ARRB1 could participate in the aggressiveness of hepatocellular carcinoma through regulating CD97 10. However, the function of ARRB1 on the development and prognosis of GBC has not been verified so far.

In this study, we utilized clinical samples and GBC cell lines to reveal the effects of ARRB1 on the GBC biological behavior. Further mechanism study revealed that ARRB1 could mediated GBC progression in a TAK1-dependent manner. Our findings may aid in the development of novel diagnostic and therapeutic strategies targeting GBC.

## Materials and methods

### Cell Lines and Cell Culture

The GBC cell lines NOZ and EH-GB1 were obtained from the Chinese Academy of Life Sciences (Shanghai, China). The cell lines SGC-996 and GBC-SD were kindly supplied by Xinhua Hospital (Shanghai, China). The SGC-996 cells were cultured in medium 1640 (Gibco, California), and NOZ, GBC-SD and EH-GB1 cells were cultured in DMEM supplemented with 10% fetal bovine serum (FBS, Gibco, USA) at 37 ℃ in humidified air containing 5% carbon dioxide (CO_2_).

### Patient Samples

Surgical GBC samples and adjacent normal specimens were obtained from 62 patients at the Affiliated Changzhou NO.2 People's Hospital of Nanjing Medical University (Jiangsu, China) from January 2014 to December 2018. All the specimens were paraffin-embedded for immunohistochemical staining or extracted total protein for western blot. This study was approved by the Institutional Ethics Committee of Nanjing Medical University and informed consent was signed before surgery. All specimens were confirmed by pathologists and the pathological diagnosis was established according to the American Joint Committee on Cancer criteria (2002).

### Immunohistochemistry

Immunohistochemistry of ARRB1 and Ki67 was performed using 5 µm paraffin sections. The sections were deparaffined in xylene and rehydrated. Antigen retrieval was performed by heating sections with citrate buffer, then removed endogenous peroxidase with 3% H_2_O_2_. Slides were washed with TBS-Triton 3 times. Non-specific binding was blocked using 5% goat serum for 30 minutes. Then sections were incubated with specific antibody (1:100). Complete the experiment and following DAB staining was done according to the instructions (KIT9730 IHC Kit, MXB, China). The scores of the staining were indexed on the intensity and percentage of the immunoreactive cells, and classified into three groups (Strong, Medium, Weak / Negative) 11. For statistical analysis, the strong group was defined as high group (n=27), and the others were defined as low group (n=35).

### Knockdown or overexpression of ARRB1 expression in GBC cell lines

Lentiviral vectors (labeled with luciferase) containing human ARRB1 short-hairpin RNA (sh-ARRB1) and negative control (sh-NC) were a gift from Professor Beicheng Sun (Nanjing University Medical School, China). Sh-ARRB1 and sh-NC sequences are presented in Supplementary Table S1. Transfected NOZ cells were verified with puromycin and RT-PCR. For overexpression of ARRB1, sequence of ARRB1 were subcloned into the lentiviral vector lentivirus vector GV248 (Corues Biotechnology, Nanjing). Recombinant lentiviruses co-transfected into 293T cells with packaging plasmids were harvested, and utilized to infect GBC-SD cells after mixing with polybrene (10 μg/ml, Sigma). Stable cells were verified by Western blotting.

### Real-time quantitative PCR

RNA was isolated from GBC cell lines using TRIzol (Invitrogen). Following DNase treatment and reverse transcription, real-time quantitative PCR (RT-PCR) was performed using one-step RT-PCR kit (TaKaRa, Japan). The specific primers used were as follows:

ARRB1 Forward: GCGAGCACGCTTACCCTTT, Reverse: CAAGCCTTCCCCGTGTCTTC; β-actin Forward: GTCTTCCCCTCCATCGTG, Reverse: AGGGTGAGGATGCCTCTCTT; TAK1 Forward: ATTGTAGAGCTTCGGCAGTTATC, Reverse: CTGTAAACACCAACTCATTGCG.

Quantification was performed by comparing the Ct values of each sample with the 2^-ΔΔCt^ method and normalization to β-actin. Values were expressed as fold induction in comparison with controls.

### Colony formation assay

The GBC cells were separately seeded and incubated in 6-well plastic plates with 500 cells for 14 days, then stained in paraformaldehyde and crystal violet successively. This assay was repeated three times. The number of clone colonies was counted and compared.

### 5-Ethynyl-2′-deoxyuridine (EdU) incorporation assay

The EdU assay was performed with the EdU detection kit (Ribo Biotechnology, Guangzhou, China) according to the manufacturer's instructions. Briefly, the cells were seeded into 96-well plates (1~5 × 10^4^ per well) and cultured overnight at 37℃ with 5% CO^2^. After 4 h of incubation in EdU medium, the cells were fixed and permeabilized, then incubated with reaction mixture, the percentage of EdU staining cells were imaged and counted under the fluorescence microscopy.

### Cell Apoptosis and Cell Cycle

Cell apoptosis was measured with the Annexin V-APC/7-AAD apoptosis kit according to the manufacturer's recommendation (Vazyme, Nanjing, China). For cell cycle analysis, the transfected NOZ and GBC-SD cells were stained with propidium iodide for 30 min, then analyzed through flow cytometry.

### Wound healing experiment

The stable cells in the logarithmic growth phase were seeded in a 6-well plate. When the cells reached almost 90% fusion, vertical wound was scratched with the head of pipette, and lightly washed with PBS, then added medium containing with 2.5% FBS. The scratch width was recorded. After cultured for 24 hours, the condition of scratch healing was measured again.

### Transwell assay

Matrigel (BD Biosciences, USA) were precoated on the upper chamber of 24-well transwells (8-μm, Millipore, USA). GBC cells (8 × 10^4^) were suspended in serum‐free medium and infused into the upper chamber. Medium supplemented with 20% FBS was applied to the lower chamber. After 24 h at 37℃ with 5% CO^2^, cells maintained in the lower surface were fixed with 4% methanol and dyed in 0.5% crystal violet. Transwell assays were independently repeated three times. The visual fields were photographed and counted under microscope.

### RNA sequencing

NOZ cells transfected with scramble or ARRB1-targeting shRNA were extracted total RNA with TRIzol respectively. Then RNA quality was tested using 2100 Bioanalyser (Agilent) and quantified with the ND-2000 (NanoDrop Technologies). Paired-end RNA-seq sequencing library was sequenced with the Illumina HiSeq xten/NovaSeq 6000 sequencer, then the KEGG pathway analysis were carried out by KOBAS (http://kobas.cbi.pku.edu.cn/home.do) 12.

### Western Blot Analysis

Total cells (or tissue) protein was extracted on ice with RIPA lysis buffer (Beyotime, China), and measured protein concentration (Beyotime, China). Equal amounts of protein samples were loaded to each lane. Then the diluents were separated by SDS-PAGE then transferred to PVDF membranes (Millipore, USA). After the blockade with 5% BSA, these membranes were incubated at different antibody diluents (β-actin (20536-1-AP), TAK1(12330-2-AP), Bcl-2 (12789-1-AP) and N-cadherin (22018-1-AP) (Proteintech Group); ARRB1(ab32099, Abcam); Vimentin (#5741), E-cadherin (#3195), ERK1/2 (#9102), and phospho-ERK1/2 (#9101s), P38(#8690), and phospho-P38 (#4511), JNK(#9252), and phospho-JNK (#4668) (Cell Signaling Technology, USA)). The immunocomplexes were visualized using electrochemiluminescence (ECL) reagent (Millipore, USA) under the appropriate exposure time according to the manufacturer's protocol. Blots were benchmarked with β-actin. The density of each band was quantified by the Image-J software.

### Nude mouse xenograft experiments

Specific pathogen-free 6-week-old female BALB/c nude mice were obtained from Model Animal Research Center of Yangzhou University and housed in the standard conditions. The NOZ cells (1 × 10^6^ / per) were subcutaneously injected into the groin of the mice (n = 6). The tumor size was measured every 5 days using a vernier caliper (tumor volume = 0.5 × width ^2^ × length). Four weeks later, all nude mice were euthanized and the tumor specimens were exteriorized and measured. Furthermore, for the tail vein metastasis model, NOZ cells (1 ×10^6^ cells/100 μl) were injected into the tail vein of mouse under anesthesia. Five weeks later, after humanly killed, the lung and liver metastatic samples were sectioned and stained with H&E to assess the extent of metastasis.

### Statistical Analysis

GraphPad Prism 6 and SPSS software were used for plotting and statistical analysis. All data was expressed as mean ± standard error of the mean. Student t-test was applied to calculate statistical significance for data sets following normal distribution. Kaplan-Meier curve was used for survival analysis. P-values less than 0.05 were considered statistically significant.

## Results

### Upregulated Immunohistochemical Staining of ARRB1 Proteins in GBC Tissues

**Figure 1:**
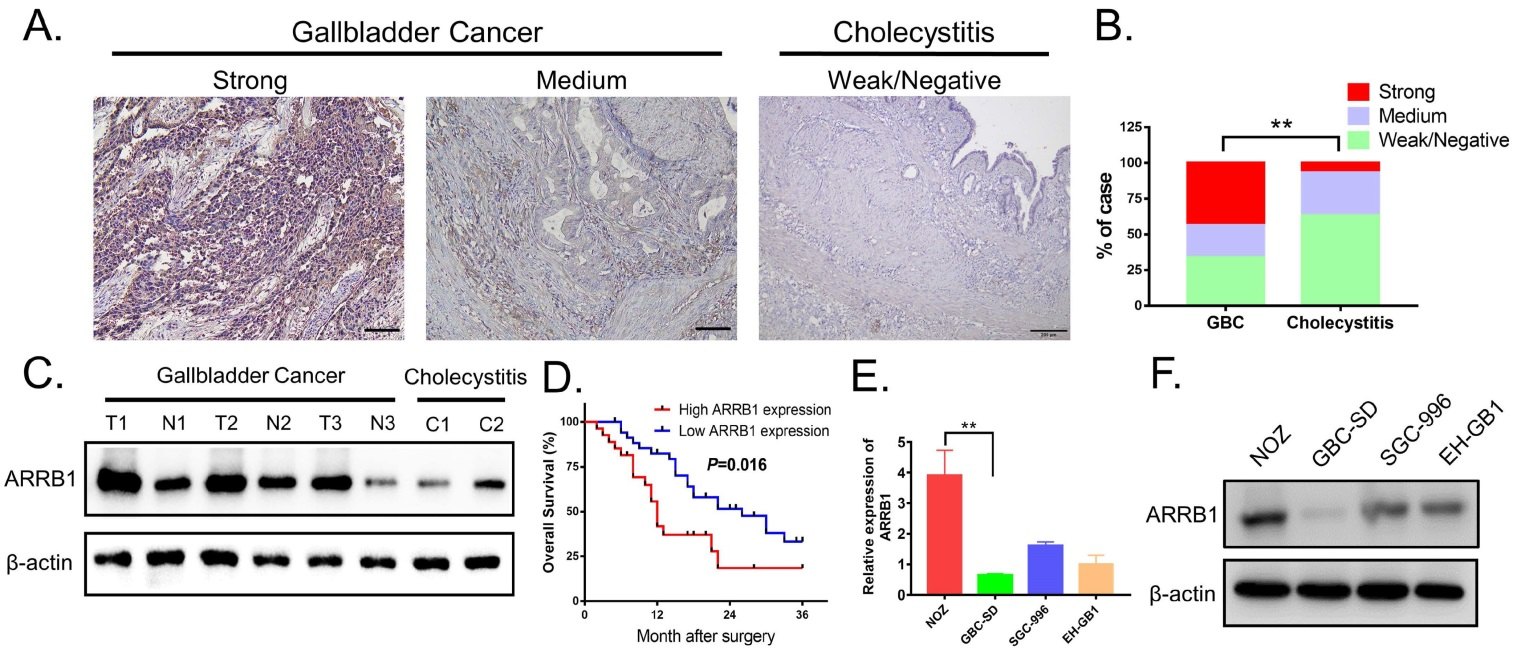
** ARRB1 expression was upregulated in gallbladder cancer tissues and clinical significance. A.** Representative photomicrographs of immunohistochemical staining of ARRB1 in GBC tissues and cholelithiasis (non-tumor samples) specimens (Magnification: 200 ×). **B.** Percentage of cases with different staining intensity of ARRB1 in GBC tissues or normal ones. **C.** ARRB1 protein level in paired GBC tissues and non-tumor tissues tested by Western Blot. **D.** Kaplan‐Meier overall survival curve of patients with GBC based on ARRB1 expression (High: n = 27, Low: n= 35).** E** and **F.** Relative mRNA and protein levels of ARRB1 in four GBC cell lines (normalized by β-actin) (*P<0.05)**.**

Assessed with immunohistochemical staining, the expression of ARRB1 tended to be higher in GBC tissues than that in cholelithiasis specimens, and ARRB1 immunoreactivity was mainly observed in the cytoplasm of tumor cells (Figure 1 A). Among the 62 GBC tissue samples, 43.55% (27/62) of cases were strong stained, 22.58% (14/62) of cases were medium stained, 33.87% (21/62) of cases were weak or negatively stained, the opposite of that only 6.67% (2/30) of the cholecystitis tissues showed strong staining of ARRB1 protein (Figure 1 B). The western blot data showed that the protein level of ARRB1 was obviously upregulated in GBC tissues compared with the nontumor counterparts and cholecystitis tissues (Figure 1 C).

### Increased ARRB1 Expression Correlates with Aggressive Clinicopathologic Characteristics and Poor Prognosis in GBC Patients

To investigate the clinical consequences of ARRB1 expression, we divided 62 GBC patients into two categories (high ARRB1 expression and low ARRB1 expression), and analyzed the correlation between ARRB1 overexpression and the pathological features as well as the disease progression of GBC. As summarized in Table 1, high levels of ARRB1 expression were significantly correlated with tumor size (***P***= 0.015) and lymphatic metastasis (***P***= 0.009). Importantly, with regard to overall survival (OS), high ARRB1 expression was correlated with worse OS rate (Figure 1 D). These findings suggested that the upregulated ARRB1 was significantly related with poor prognosis of GBC.

### Knockdown of ARRB1 regulates proliferation, migration, and invasion of GBC cell *in vitro*

**Figure 2:**
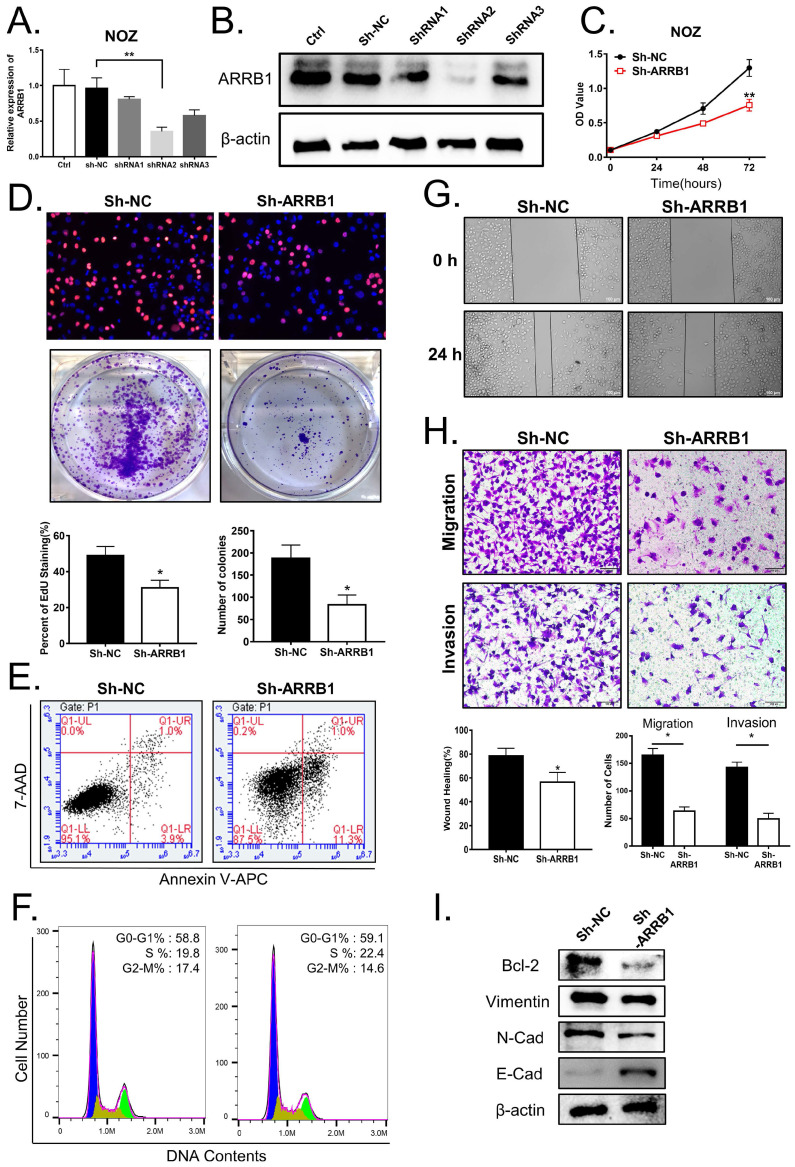
** ARRB1 promoted GBC cell growth, migration and invasion of GBC cells. A** and** B.** Knockdown efficiencies of ARRB1 with shRNA1, shRNA2 and shRNA3 in NOZ cells were determined by RT-PCR and Western blot, controlled with sh-NC. **C.** Cell viability tested by CCK‐8 in NOZ cells transfected with sh‐ARRB1 or sh‐NC. **D.** Cell proliferation analyzed by colony formation and EdU assay in NOZ cells treated as indicated. **E.** Flow cytometry analysis of cell apoptosis in ARRB1 knockdown NOZ cells, compared with sh‐NC. **F.** Flow cytometry of cell cycle in NOZ cells with down-regulated ARRB1.** G.** Wound healing assay comparing the migration ability between ARRB1 knockdown and negative control NOZ cells. **H.** Transwell assays were used to detect the changes of cell migration and invasion capacities in NOZ cells after transfection. (Magnification: 200 ×, Scale bar: 100μm). **I.** Western blot for Bcl-2, Vimentin, E‐cadherin and N‐cadherin expression in different treated NOZ cells. All data were shown as mean ±SD (*P<0.05). All experiments were repeated at least three times.

To further elucidate the biological role of ARRB1 in GBC, we first detected the expression of ARRB1 in GBC cell lines and found that ARRB1 was higher expressed in NOZ and lower expressed in GBC-SD cell lines compared with others respectively (Figure 1 E, F). As NOZ cells showed higher ARRB1 expression, we transfected them with lentiviral shRNAs (shRNA-1, shRNA-2 and shRNA-3) specially against ARRB1 and the negative control (sh-NC). The interference efficiency of shRNAs were tested by qRT‐PCR and western blot (Figure 2 A, B). Because of more effective suppression, shRNA-2 was selected for further experiments.

Firstly, as shown in Figure 2 C, D, the results of the CCK-8 and EdU assay demonstrated that the ARRB1 knockdown notably inhibited the NOZ cells proliferation. Moreover, the colony formation assay showed the clonogenicity of NOZ decreased significantly due to the block of ARRB1 (Figure 2 D). As cell apoptosis and cell cycle arrest has been widely reported to affect the proliferation of tumor cells, we next detected the influences of ARRB1 on cell apoptosis and cell cycle distribution. The percentage of apoptosis in down-expressed ARRB1 NOZ cells was markedly increased compared to that of control cells (Figure 2 E, and Figure S1 A), while the cell cycle analysis showed no statistical differences (Figure 2 F, and Figure S1 B). Secondly, to investigate the role of ARRB1 on GBC cell motility, we performed wound healing and transwell migration assays, and found that loss of ARRB1 expression significantly suppressed random motility, migration, and invasion of NOZ cells (Figure 2 G, H). It also significantly altered the level of the related apoptosis protein (Bcl-2), as well as the epithelial-mesenchymal transition (EMT) proteins (Vimentin, E-cadherin and N-cadherin) (Figure 2 I). Collectively, these results strongly implicated that ARRB1 was an oncogenic gene in GBC cells.

### ARRB1 promotes tumor progression *in vivo*

**Figure 3:**
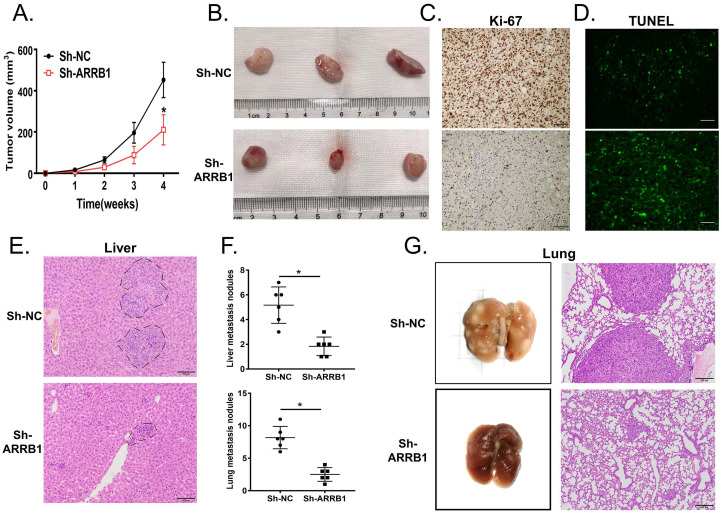
** ARRB1 promoted tumor growth and metastasis in GBC cells *in vivo*. A.** The subcutaneous tumor growth curve of xenograft mice injected NOZ with Lv-Sh-NC or Lv-Sh-ARRB1 (n=6). **B.** The tumors of mice xenograft executed at day 28 after injected with cells mentioned above. **C.** Immunohistochemistry staining of Ki67 in mice subcutaneous tumors after silencing the ARRB1. **D.** TUNEL assay was carried out for revealing cell apoptosis in different ARRB1 expression of NOZ cells. **E.** Tail vein xenograft models were established to show the metastatic role of ARRB1 *in vivo*, and H&E staining of ARRB1 knockdown and NC groups were shown representatively (n=6). **F.** Metastatic tumors in liver and lung were identified and compared with each NC group.** G.** Lung metastasis specimens observed by autopsy and H&E staining at Day 35 after cells injected (*P< 0.05, Magnification: 200 ×, Scale bar: 100μm).

The results above suggested that ARRB1 plays important roles in GBC tumor progression *in vitro*. To extend our investigations *in vivo*, NOZ cells were injected into BALB/c nude mice to construct the xenograft model. Tumors expressing normal level of ARRB1 grew rapider than NOZ-shARRB1 ones. After 28 days, we observed the tumor size distinctly diminished on account of ARRB1 downregulation (Figure 3 A, B). Confirmed with *in vitro* experiments, lower Ki-67 expression was tested in shARRB1 group than that in sh-NC group by IHC (Figure 3 C and Figure S1 C). Consistently, TUNEL staining showed that suppressed ARRB1 could stimulated the apoptosis of tumor cells (Figure 3 D and Figure S1 D).

The metastatic model of the nude mice was established by the tail intravenous injection of NOZ cells. The number and size of liver metastatic nodules per nude mice was significantly declined in ARRB1 knockdown group compared with control group (Figure 3 E, F). Furthermore, the pulmonary metastasis lesions in group injected with NOZ-shARRB1 cells were smaller and fewer assessed both by visual inspection and microscope (Figure 3 F, G). All observations of *in vivo* experiment distinctly suggested that ARRB1 could promote GBC tumorigenesis and metastasis.

### ARRB1 silencing restrains the expression of TAK1 and inhibits MAPK pathway in GBC

**Figure 4:**
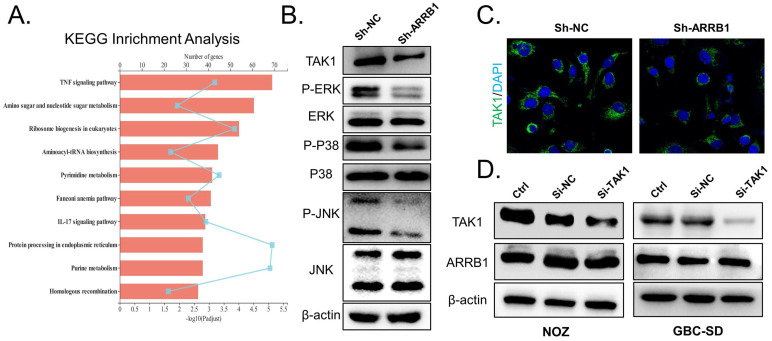
** Loss of ARRB1 restrained TAK1 and attenuated pathway activity in GBC. A.** Statistics for the KEGG pathway analysis of differentially expressed transcripts identified by RNA-seq between the shARRB1 and sh-NC groups. **B.** Western blot for TAK1 and downstream MAPK signaling expression in different treated NOZ cells. **C.** Immunofluorescence analysis of TAK1 levels in different groups. **D.** GBC cell lines were transfected with the si-TAK1 and harvested for examining the expression of TAK1 and ARRB1(*P<0.05).

Multiple past studies have shown that ARRB1 regulates specific cellular functions by interacting with specific partner proteins, including PI3K and MAPK pathway 7. To explore the downstream regulatory mechanisms by which ARRB1 acts in GBC, we exploited RNA sequencing to compare the transcriptome of sh-NC and sh-ARRB1 groups. Many genes were differentially expressed when ARRB1 was depleted (Figure S1 E). Further KEGG analysis identified that the most significantly regulated pathway in the ARRB1 knockdown GBC cells was TNF signaling (Figure 4 A). Recent work has demonstrated that TNF-α could directly induce hepatic ARRB1 expression and enhance hepatocellular carcinogenesis via ARRB1-AKT interaction by binding to boost Akt phosphorylation 13. Additionally, in intestinal epithelial cells of colitis, TNF-α/ARRB1-dependent signaling in hematopoietic and non-hematopoietic cells differentially regulates colitis pathogenesis in modulating MAPK pathways 14. Preliminary literatures combined with our results indicated that ARRB1 may be a critical TNF signaling regulator in GBC. To verify the hypothesis, we performed RT-qPCR to confirm the expression of these candidate genes with a change of greater than 2.5-fold involved in TNF signaling. The results showed that shRNA-mediated ARRB1 could regulated the expression levels of TAK1 (MAP3K7), TRAF3 and NIK (Figure S1 F, G). Notably, TAK1 was the most strongly downregulated gene when ARRB1 was knocked down in GBC cells. Subsequently, western blotting and immunofluorescence (IF) staining both revealed that TAK1 protein level was dramatically repressed after ARRB1 depletion (Figure 4 B, C). Synchronously, same tendency was found in the phosphorylation of Erk, p38 and JNK (Figure 4 B). After treated with siRNA-TAK1(sc-36606), the expression of TAK1 in NOZ and GBC-SD cells reduced, while the ARRB1 level was not affected by this treatment (Figure 4 D). These findings suggested that ARRB1 positively coordinates the level of TAK1 to activate the TNF/MAPK signaling pathway.

### ARRB1 controls GBC biological functions partly through TAK1

**Figure 5:**
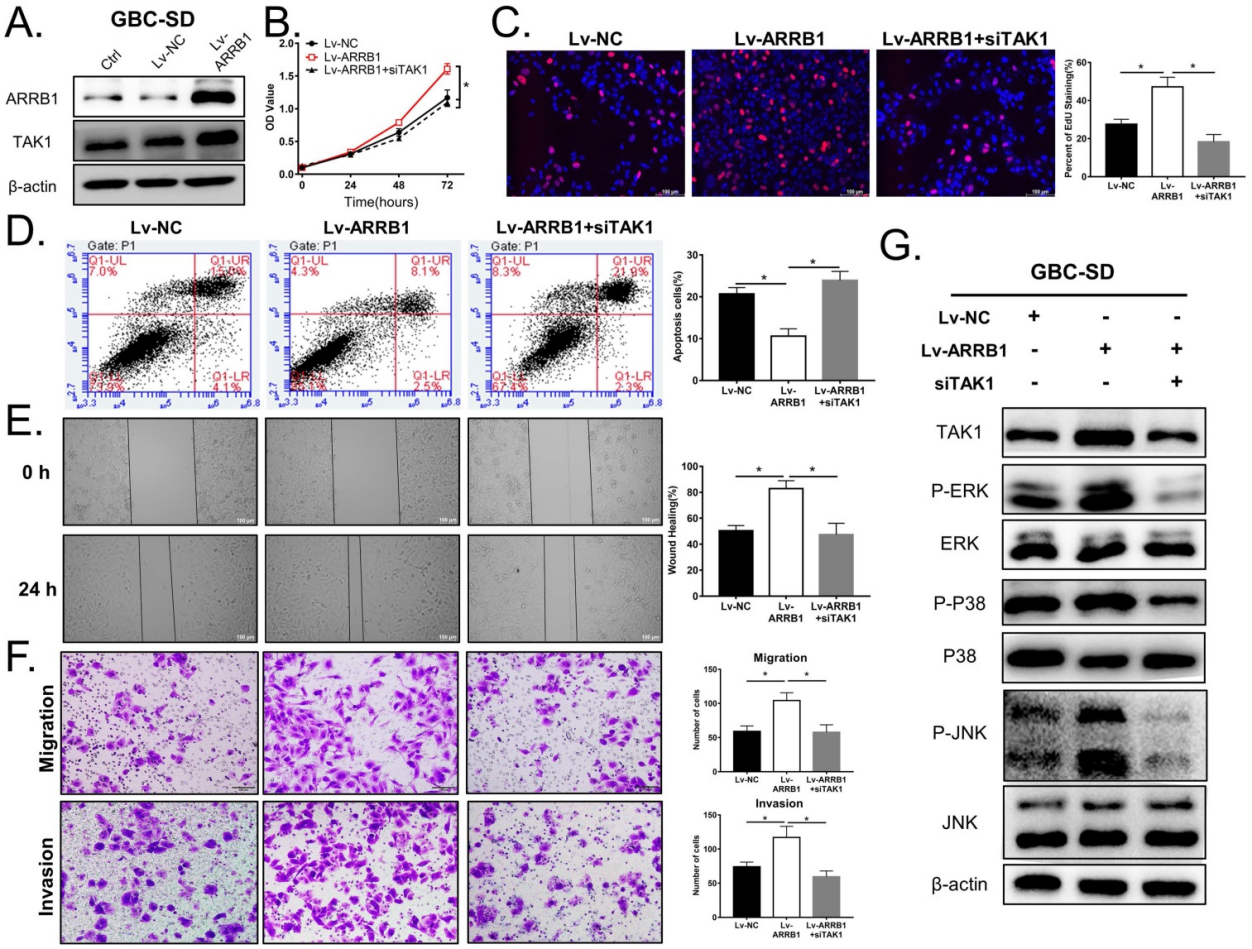
** The oncogenic role of ARRB1 was partly based on its regulation of TAK1. A.** GBC-SD cells transfected with the Lv-ARRB1 and Lv-NC were harvested for Western blot.** B and C.** The cell vitality of NC, Lv-ARRB1+siNC (equivalent to Lv-ARRB1), Lv-ARRB1+siTAK1 treated GBC-SD cells were respectively measured by CCK8 and EdU assay.** D.** The cell apoptosis rates of differently treated GBC-SD cells were assured by flow cytometer using Annexin V-APC (X axis) and 7-AAD (Y axis) staining. **E and F.** The cell migration and invasion abilities of the above cells were respectively measured by wound healing and transwell assay.** G.** Western blot analysis of the expression level of TAK1 and related downstream MAPK pathway protein (p-ERK, p-P38 and p-JNK) in the above cell lines, normalized by β-actin expression. Data were from at least three independent experiments with the way of Mean ± SD (*P<0.05).

To investigate whether TAK1 is indeed required for the biological effects of ARRB1 depletion in GBC, we designed the following rescue experiments. First, Lv‐ARRB1 lentivirus was used to upregulate ARRB1 expression in GBC-SD cell line, which exhibited low‐expression level of ARRB1. The raised expression of ARRB1 was verified by western blot detection, and the expression of TAK1 increased accordingly (Figure 5 A).

Functionally, ectopic expression of ARRB1 in GBC-SD cells evidently promoted cell viability and proliferation, which could be partially rescued by TAK1 suppression (Figure 5 B, C). As for apoptosis, the percentage of apoptosis showed significant differences in ARRB1 overexpressed GBC cells in comparison with the Lv-NC or adding si-TAK1 synchronously (Figure 5 D). In addition, upregulation of ARRB1 significantly stimulated the metastasis of GBC-SD cells, as demonstrated by wound healing and transwell assays, which could be counteracted by knocking down TAK1 (Figure 5 E, F). As shown in Figure 5 G, the same trend in the downstream of TAK1/MAPK signal path was demonstrated. Taken together, all findings supported that TAK1, as a downstream pathway of ARRB1, mediated vital effects of ARRB1 on the GBC cell proliferation, apoptosis, migration, and invasion.

## Discussion

Gallbladder carcinoma is a highly lethal and aggressive disease, and the therapeutic outcome of non-operative treatment for GBC is not satisfactory. The poor prognosis of this disease is also due to early metastasis and delayed diagnosis, as the 5-year survival rate is less than 6% 15. Therefore, it is urgent to increase our understanding of the pathogenesis and molecular mechanism of GBC for screening out more effective therapeutic target.

ARRB1 is a member of β-arrestin family, mostly recruited associated with signaling desensitization of G-protein coupled receptors (GPCR), to achieve spatiotemporal specificity of different signaling complexes 16, 17. ARRB1 could act as cytosolic, nuclear scaffold or signal transducer, controlling in multifaceted signaling processes, such as cell proliferation, metastasis and drug resistance 18. For ovarian cancer, the recruitment of ARRB1 showed the remarkable ability as a checkpoint converging pathway on β-catenin signaling to promote the invasion and metastasis of ovarian cancer cells 19. In glioblastoma, knockdown of ARRB1 decreases cell viability, metastasis and glycolysis by suppressing Src signaling 20. Our data firstly showed that ARRB1 expression was upregulated in human GBC tissues compared with normal gallbladder epithelium tissues. In addition, the related poor clinical characteristics suggested that ARRB1 assuredly played an important role in progression of GBC. Through a series of *in vitro* and *in vivo* assays, we confirmed that inhibition of ARRB1 restrained the GBC cell proliferation, metastasis and tumor growth. Moreover, RNA sequencing and our following research showed that ARRB1 deletion decreased the activation of TNF/MAPK signaling pathway in GBC. TNF-α is a key inflammatory cytokine responding to chronic inflammation which is a major carcinogenic mechanism of gallbladder cancer 21. The promote relationships between TNF-α and Vascular Endothelial Growth Factor-C (VEGF-C), ARRB1 and VEGF-C has been confirmed in many diseases including pulmonary hypertension, colitis and gallbladder cancer 22-24. To further clarify the downstream signaling mechanism involved by ARRB1 in TNF-α/MAPK, we used ARRB1-overexpressed GBC-SD cells and siTAK1 to further demonstrate that ARRB1 positively regulated the TAK1/MAPK signaling pathway. TAK1, thought to mediate much of the intracellular actions of TNF-α, is closely involved in inflammation-related diseases 25. Our study complemented the inflammation-carcinogenic mechanism of ARRB1/TAK1, and firstly verified the important role of ARRB1/TAK1 in the regulation of proliferation, apoptosis and metastasis in GBC.

In this study, we conclude that ARRB1 regulates GBC progression by modulating the TAK1/MAPK pathway. However, emerging data shows that ARRB1 may be driven by many non-GPCR pathways, such as endothelin-1 TGF-β and VEGFR 26, 27. The carcinogenic mechanism of inflammation linked to TGF-β or TNF-α and its regulation of ARRB1 in GBC or other cancers still needs to be fully elucidated. According to the results of RNA sequencing, we focused on and verified the ARRB1/TAK1/MAPK signal path. Interestingly, in portal hypertensive gastropathy, ARRB1 was confirmed to regulated ER stress-induced mucosal epithelial apoptosis through suppression of the TNF-α/p65 signaling pathway activation 28. Synchronously, ARRB1 also mediated the development of nonalcoholic fatty liver disease through TRAFs, which is another important member of TNF-α pathway 29. The specific mechanism of ARRB1 participation in the downstream pathway of TNF-α/MAPK and whether it forms a network system with other pathways, such as PI3K and NF-κB, are worthy of further study. Focus on clinical translational applications; we have noticed that the TAK1 inhibition-NG25 could significantly inhibit tumor cell growth in different cancers 30, 31. Combined with our results, it is of great research value to exploit ARRB1 or its downstream TAK1 as a therapeutic target for GBC, we speculate that inhibition of ARRB1 or TAK1 with specific molecule compounds may be effective for GBC treatment.

Altogether, our study firstly investigated that ARRB1 was related to the poor prognosis in GBC, and functioned as an oncogenic gene. Knockdown of ARRB1 restrained the cell proliferation by promoting apoptosis, and inhibit the migrative and invasive ability of GBC cells *in vitro* and *in vivo*. Moreover, the tumor-promoting effect of overexpression ARRB1 may be partially related the activations of TAK1/MAPK axis. Therefore, ARRB1 may serve as a potential target for cancer diagnosis of GBC and related therapies.

## Supplementary Material

Supplementary figures and tables.

## Figures and Tables

**Table 1:** Clinical characteristics and ARRB1 expression in patients with GBC.

	No. of cases	ARRB1 expression		*P*
		High	Low	
All patients	62	27	35	
Age				0.492
<60	26	10	16	
>60	36	17	19	
Gender				0.420
Male	17	6	11	
Female	45	21	24	
Tumor size				**0.015**
<4cm	36	11	25	
>4cm	26	16	10	
TNM stage				0.052
I and II	27	8	19	
III and IV	35	19	16	
Lymphatic metastasis				**0.009**
Yes	21	14	7	
No	41	13	28	
Histology differentiation				0.687
High	19	9	10	
Moderate/Low	43	18	25	

The data in this table is analyzed using chi-square test. * indicates ***P*** value < 0.05

## Availability of data and materials

The data analyzed in this study are available from the corresponding authors with a reasonable request.
