# Supplementary material for: ARRB1 Drives Gallbladder Cancer Progression by Facilitating TAK1/MAPK Signaling Activation
Source: J Cancer. 2021 Jan 30;12(7):1926–35. doi: 10.7150/jca.53325 (PMC7974532; doi:10.7150/jca.53325)
Supplement: Supplementary file 1 — Supplementary figures and tables. [file jcav12p1926s1.pdf]

Supplementary Table S1. Summary of shRNA and siRNA Oligos

Name	Oligo Sequence
ARRB1 shRNA 1	CCGGGGAGAGTCTATGTGACGCTGACTCG AGTCAGCGTCACATAGACTCTCCTTTTTG
ARRB1 shRNA 2	CCGGGCAGGAACGCCTCATCAAGAACTCG AGTTCTTGATGAGGCGTTCCTGCTTTTTG
ARRB1 shRNA 3	CCGGTGGAACTGCCCTTCACCCTAACTCG AGTTAGGGTGAAGGGCAGTTCCATTTTTG
Sh-NC	GGATCCGTTCTCCGAACGTGTCACGTTTCA AGAGAACGTGACACGTTCGGAGAATTTTTT
TAK1 siiRNA	GUAAACACCAGCUCAUGGCAUGAGC

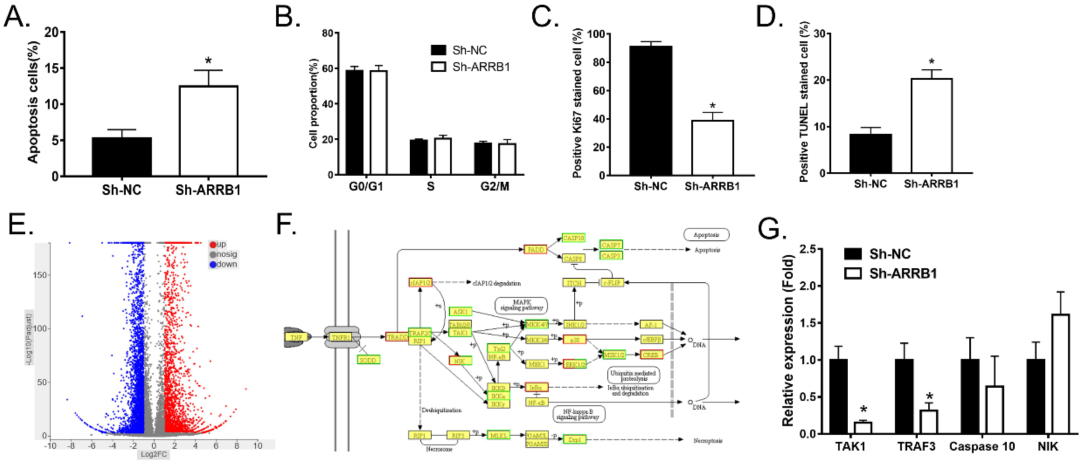

Figure Supplementary 1.

A. Percentage of apoptotic cells in ARRB1 knockdown NOZ cells, compared with sh-NC group. **B.** Cell cycle percentage distribution in cells mentioned above. **C and D.** The positive stained cells of Ki67 and TUNEL in subcutaneous tumors after silencing the ARRB1 and control. **E.** Volcano plot (fold change > 2.5 and P value < 0.01) detected by RNA-seq analysis. **F.** TNF signaling pathway showed upregulated (red) and downregulated (green) differentially expressed genes between the sh-NC and shARRB1. **G.** The mRNA levels of the aforementioned genes (TAK1, TRAF3, Caspase 10 and NIK) were measured by RT-PCR**.** * $P < 0.05$.
